# Assessment of the Possibilities for the Use of Selected Waste in Terms of Biogas Yield and Further Use of Its Digestate in Agriculture

**DOI:** 10.3390/ma15030988

**Published:** 2022-01-27

**Authors:** Marcin Niemiec, Jakub Sikora, Anna Szeląg-Sikora, Zofia Gródek-Szostak, Monika Komorowska

**Affiliations:** 1 Faculty of Agriculture and Economics, University of Agriculture in Krakow, Mickiewicza 21, 31-120 Kraków, Poland; monika.komorowska@urk.edu.pl; 2 Faculty of Production and Power Engineering, University of Agriculture in Krakow, ul. Balicka 116B, 30-149 Kraków, Poland; Jakub.Sikora@urk.edu.pl (J.S.); Anna.Szelag-Sikora@urk.edu.pl (A.S.-S.); 3 Institute of Management and Production Engineering, Cavalry Captain Witold Pilecki State University of Małopolska in Oświęcim, Maksymiliana Kolbego 8, 32-600 Oswiecim, Poland; 4 Department of Economics and Enterprise Organization, Cracow University of Economics, ul. Rakowicka 27, 31-510 Kraków, Poland; grodekz@uek.krakow.pl

**Keywords:** biogas, renewable energy sources, organic fraction of municipal waste, sewage sludge, digestate, management

## Abstract

The utilization of municipal waste and sewage sludge as a source of energy is technically very difficult due to high variability of their physical and chemical properties. The aim of this study was to evaluate the efficiency of the conversion of biomass contained in the whitewater fraction of municipal waste and sewage sludge by means of methanogenesis. The second objective was to assess the chemical composition of the digestate in the context of its use for fertilizer purposes. The whitewater fraction of municipal waste and sewage sludge was subjected to methanogenesis under static experimental conditions, according to DIM DIN 38414 methodology. The methanogenesis of concentrated substrates used in agricultural biogas plants was taken as a reference to evaluate the efficiency of the process. The organic fraction of the municipal waste was characterized by approximately 30% lower value of the soluble COD, with a comparable level of total COD compared to other materials. The total biogas yield, i.e., 404 dm^3^ per 1 kg of dry weight of the batch, was measured in the facility with sewage sludge. In COD value, this is 0.232 dm^3^·g O_2_ COD. In the case of corn, these values were, respectively, 324 dm^3^ and 0.193, and for the organic sub-sieve fraction of municipal waste, 287 dm^3^·kg^−1^ dw or 0.178 dm^3^·g O_2_ COD, respectively. The type of fermented material did not affect the intensity of biogas production. The maximum level of biogas production occurred between the 13th and 15th day of the process. The digestate obtained in the process of methanogenesis of corn silage and the organic fraction of municipal waste was characterized by good parameters in terms of possible use for fertilization purposes.

## 1. Introduction

The development of zero-waste production technologies is a strategic element of all types of human activity under sustainable development [1]. Product manufacturing technologies should take into account the quality requirements related to resistance to environmental factors, regardless of the type of raw material [2,3]. Waste generated in one production sector can be input material in the production of other goods. Waste materials can be used both directly as raw materials and through the use of the elements contained in them as a source of plant nutrients [4,5,6,7]. The re-use of plant nutrients contained in rainfall allows the biogeochemical cycle of elements to be limited to a single farm or economic region. This reduces the demand for elemental fossil raw materials, e.g., phosphorus or potassium, and for production ingredients, which require a large amount of energy, such as nitrogen [8]. The production of energy from biomass or organic waste is gaining importance in developed countries. The greenhouse gas emission reduction policy, as well as the need to diversify energy sources, has become the foundation for the development of biomass fuel production technology [9,10,11]. Biomass, including that from waste sources, can be converted in different ways; however, methanogenesis is one of the most widely used processes in the energy transformation of organic matter [12,13]. Thus, obtained gas can be used as a heat source or as a substrate for the production of electric power [14,15]. The resulting biogas can be problematic due to the high number of impurities that pose an environmental hazard when burned directly. This can be avoided with one of the most advanced technologies for biogas utilization, i.e., dry reforming, which converts pure biogas to hydrogen and carbon monoxide [16,17,18]. Biogas purification and upgrading methods increase the energy efficiency of biomass utilization for energy purposes, however due to the high number of impurities, biogas must be burned in dedicated installations. The products of dry reforming of biogas are versatile fuels [19]. Contemporary researchers are increasingly pointing to the possibility of creating hybrid plants for biomass conversion. Jung et al. [20] indicate that for wastes of varying composition, such as animal manure, beneficial results can be obtained when methanogenesis is combined with alcohol production and fertilizer production. In most modern biogas plants, the input material is mainly mixtures of manure and energy crops, mostly corn silage due to the high volume of biomass in these plants and the possibility of storing the raw material, as corn silage is microbiologically stable for an extended time. The production of energy crops, however, is associated with the need to use large amounts of energy for agrotechnical treatments, the production and application of fertilizers and plant protection products, as well as the harvesting and substrate preparation processes. Therefore, the use of cultivated energy crops can result in low energy efficiency and significant emission of greenhouse gases per unit of energy obtained. Therefore, recently more and more focus has been placed on the possibility for the use of various types of waste as a methane production substrate [21,22]. Sewage sludge and municipal waste are interesting sources of energy due to the high content of organic carbon. Traditional methods of their utilization involve landfilling or, in the case of municipal waste, composting, which are related to the emission of greenhouse gases and odors. In terms of environmental efficiency, the thermal treatment of this waste is a slightly better solution; however, the process is inefficient both from the energy and environmental point of view. From the point of view of energy efficiency and environmental impact, production optimization is an integral part of all modern quality management systems in primary production [23,24,25,26]. The energy extraction from waste is a strategic element of sustainable waste management [27,28,29,30]. The ecological aspect of the processing of waste biomass by methane fermentation is related not only to the obtaining renewable energy, but also to the rational disposal of this waste, as to well as the reduction of greenhouse gas emissions resulting from waste storage and the production of conventional energy [31,32,33,34]. The by-product of methane fermentation is digestate, which, if introduced into the soil, can be a valuable source of elements for plants. The use of digestate for fertilization increases the level of carbon sequestration in soil resources and supports the effective management of soil fertility [35,36,37]. Structural and organizational changes in agriculture have led to a reduction in the use of organic materials for fertilization. In research related to the use of food industry waste for fertilization, special attention is paid to phosphorus, the resources of which will be exhausted by the end of the 21st century [38]. The use of this waste in biogas production and then using the digestate for fertilization can be an important link in the circulation of elements in agroecosystems, as part of rational agricultural production methods. Improving soil properties and enriching it with macro-, and microelements reduces the demand for the use of mineral fertilizers, the production of which is also associated with the emission of greenhouse gases. The digestate resulting from the methane fermentation process is devoid of pathogens: Salmonella and Escherichia coli bacteria, viruses, fungi, and parasites. Pathogen disappearance rate and effectiveness are influenced by parameters such as pH, temperature, time, and the level of volatile fatty acids. The sanitary aspect is critical when the digestate is to be used for fertilization [39]. There is an increased risk of excessive accumulation of trace elements in the case of animal aquaculture and marine ecosystem products [40,41]. An important instrument supporting the development of waste energy production methods are the EU legislation acts, the most important of which are: the Landfill Directive [42], requiring the reduction of biodegradable waste bound for landfills, and the Waste Framework Directive [43]. 

Under the Regulation of the European Parliament and the Council No. 1069/2009/EC [44], animal waste is an animal by-product. The regulation distinguishes 3 categories of waste according to the degree of human and environmental hazard. Waste classified as category 1 must undergo thermal treatment. Categories 2 and 3 are waste that can be used to produce biogas. Human consumption poultry slaughter waste was classified as category 3. According to the law, it can be biogased after prior pasteurization at 70 °C for 60 min. The Act of 14 December 2012 on waste [45] does not include the provisions of the regulation, except for products that are “waste intended for storage in a landfill, or incineration, or for use in a biogas plant, or in a composting plant under this regulation.” Therefore, animal waste intended for disposal, e.g., in a biogas plant, is still considered waste in the light of the Act. 

The research aimed to determine the quantity and quality of the released biogas during methane and sewage sludge fermentation. The research goal was first to determine the suitability of the under-sieve fraction of municipal waste sewage sludge for methane fermentation by analyzing the quantity and quality of the obtained biogas. Secondly, the research was to assess the chemical composition of the post-fermentation digestate.

## 2. Materials and Methods

The adopted research objective was delivered based on a laboratory experiment, which comprised methane fermentation of the organic sub-sieve fraction of municipal waste and sewage sludge. Corn silage, i.e., the raw material most often used for biogas production, was used as a reference to compare the results of the experiments. The sub-sieve fraction was obtained from a sorting line used in a waste management plant that collects, segregates, disposes of, and utilizes municipal waste. Initial preparation of the input material used in the methanogenesis included separating the organic fraction with granulation under 5 cm. The municipal waste fraction was separated on drum sieves with 5 cm × 5 cm mesh. Its morphological composition was determined based on the obtained sub-sieve fraction and 10 component groups were distinguished. Only the organic fraction of the analyzed waste was fermented. The morphological composition of the sub-sieve waste fraction is shown in Table 1. 

The organic fraction of municipal waste used in the research consisted of potatoes, fruit, vegetables, bread, paper, meat scraps, and bones. Due to the nature of the material used in the process, it also contained mineral elements and small-particle fragments of plastic, glass, and other undetermined waste. The sewage sludge was collected from the Sewage Treatment Plant in Krosno, Poland at the turn of September and November 2020. It is a mechanical and biological treatment plant with a capacity of 35,410 m^3^·day^−1^, with chemical precipitation of phosphorus and a sewage sludge processing line. The facility collects sewage from Krosno and surrounding communes. Table 2 presents selected parameters of the materials used as input for the methanogenesis process. The reference point for the experiments conducted was corn silage.

The prepared materials were placed in 2 dm^3^ reactors. A 2 kg batch contained 1559.3 g of dry and ground sub-sieve fraction and 441 mL of H_2_O. The prepared fermenters (Zakłady Automatyki Rotametr, Gliwice, Polska) were placed in a chamber with temperature regulation. Next, the samples were subjected to static methane fermentation following the DIM DIN 38414 methodology. It consisted of a single introduction of substrates into the fermentation chambers, after which the process was carried out until its completion. The fermentor environment was maintained at a pH of 5.8–6.2. The pH was maintained by adding an inoculum. The methane fermentation process lasted 32 days. The gas produced in the methane fermentation was collected in tanks of different volumes for each fermenter. The NANO SENS 60 m (NANOSENS Sp. z o.o., Poznań, Polska) was used to measure the moisture of the produced biogas and to determine its chemical composition. The result parameters of the process were read daily at the same time using a measuring system and automatically saved on a hard disk.

To assess the potential of the resulting digestate as a source of plant nutrients, the chemical composition of both the feed and digestate was determined. The content of nitrogen, organic carbon and other macronutrients: N, Ca, P, Na, K, and Mg was determined in the samples of the tested waste. Also, the content of trace elements: Cu, Fe, Zn, Mn, Ni, Pb, Cr, and Cd was determined. Laboratory sample was collected from each object for analysis. The laboratory sample size for fermentation input products was 1000 g, while for the digestate, the laboratory sample was 200 g. The laboratory samples were dried at 65 °C, homogenized, and subjected to wet mineralization in a closed system using microwave energy. Multivawe 3000 system by Anton Paar, Graz, Austria was used for mineralization.

The analytical weight was 0.5 g d.w. The concentration of the examined elements in the obtained solutions was determined by atomic emission spectrometry using the 7600 DV spectrometer by Perkin Elmer, Boston, MA, USA. The wavelengths used to determine the concentration of the tested elements and the determination limits of the methods used are presented in Table 1. The content of total nitrogen and organic carbon was determined by elemental analysis using the Vario Max Cube apparatus by Elementar Analysensysteme GmbH, Langenselbold, Germany. The IEA-V-10 certified reference material was used to verify the validity of the analysis. Table 1 shows the results of the reference material analyzes and the estimated recovery value, based on 4 replicates. 

## 3. Results and Discussion

The methanogenesis process is sensitive to environmental parameters such as pH, hydration, carbon to nitrogen ratio, as well as the content of toxic organic compounds and heavy metals. Therefore, in the case of materials that are difficult to biogasify, various mixtures are used to optimize the process environment [46]. For some types of waste, such as sewage sludge or municipal waste, the introduction of additional materials can be problematic in terms of logistics or environmental protection. Municipal solid or liquid waste treating facilities are generally remote from agricultural areas where agricultural biomass for co-fermentation can be obtained. In addition, sewage sludge or municipal waste can be contaminated with organic compounds or heavy metals, which can disqualify the use of digestate for agricultural purposes. The optimal value of the carbon to nitrogen ratio for methanogenesis ranges from 10:1 to 30:1. For sewage sludge, the value of this parameter was 8.887 for municipal waste 14.54, and corn silage—13.96 (Table 3).

Optimum carbon to nitrogen ratios will vary as per the materials used. Romano and Zhang [47] found that the most favorable carbon to nitrogen ratio in the fermentation of onion juice and sewage sludge is 15. For the batches used in own research, the values of this parameter were within the optimal limits, however, significant differences between the individual components were found. 

The factor limiting the effectiveness of the sewage sludge methanogenesis process is the large amount of high-molecular organic compounds that are poorly degradable in anaerobic decomposition [48]. To optimize the process, various technological additives such as glucose can be used to increase the efficiency of the process, or the sludge can be pre-treated with heat. Although the addition of easily degradable compounds or thermal treatment of the batch is effective in terms of process efficiency, their use may not increase environmental efficiency as it significantly increases the carbon footprint of the methanogenesis process [49]. 

To determine the biogas yield, the sum curve method was chosen, in which the portion of the obtained gas from each day is added together to obtain the lines of biogas release from the substrates. This approach allows for observing how the digestion of the biomass in the digester bed proceeded and how the nutrients contained in the organic matter were available to the methane bacteria.

To illustrate the variability of results of the biogas fermentation, variance was used. It is the basic measure that describes the variability of the results. The variance tells how much variation there is in the results in a given set of results (the variable). In other words, variance reveals how the results are more concentrated around the mean: whether there are small differences between the mean and individual results, or whether the difference of individual results from the mean is large.

**Figure 1:**
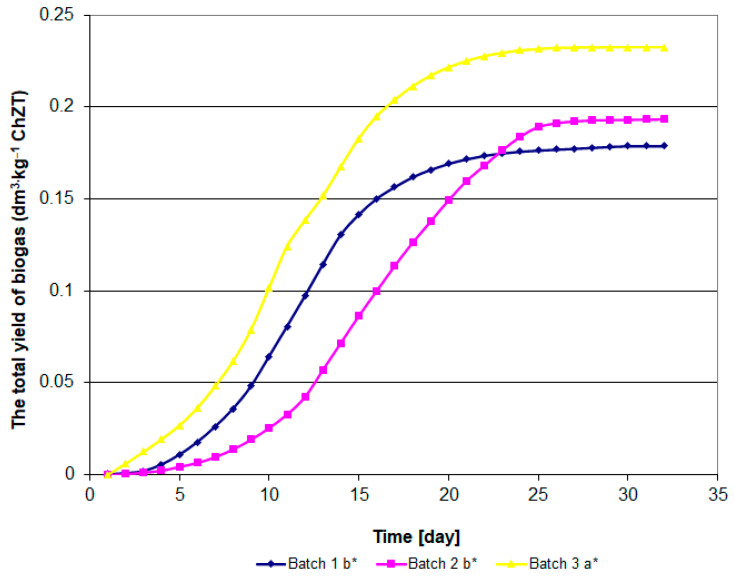
Biogas yield per COD value of input ma terials (dm^3^ biogas·g O_2_).

**Figure 2:**
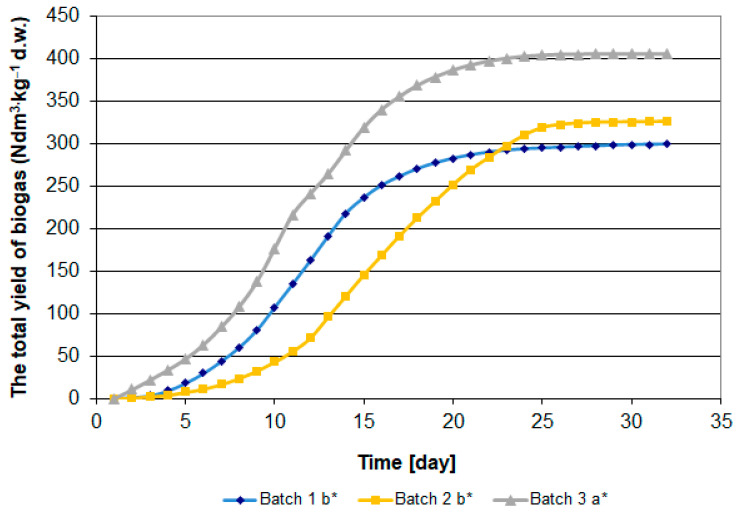
Biogas yield per dry mass of input materials (dm^3^ biogas kg dw of material). Batch 1—sludge, Batch 2—organic fraction of municipal waste, Batch 3—corn silage. * Different letters mean statistically significant differences at the significance level *p* = 0.05.

The analysis of variance was conducted in a system with a single qualitative predictor. To illustrate significant differences between biogas yields, Tukey’s test was used as one of the most commonly used tests for comparing pairs of means, especially when sample sizes vary. In Figure 1 and Figure 2, the letters (a, b) indicate homogeneous groups in terms of biogas release from the substrate during fermentation, based on the studentized range distribution. Tukey’s method is more conservative than e.g., Fisher’s Least Significant Difference (LSD) test, but less so than Scheffé’s test. The experimental error rate for all pairwise comparisons remains at the error rate for the set, which means that if a statistical significance level of α = 0.05 is assumed for the ANOVA test, the same statistical significance level will be used for all pairwise (sample) comparisons. This procedure is used when the assumption of equality of variance across samples is met.

**Figure 3:**
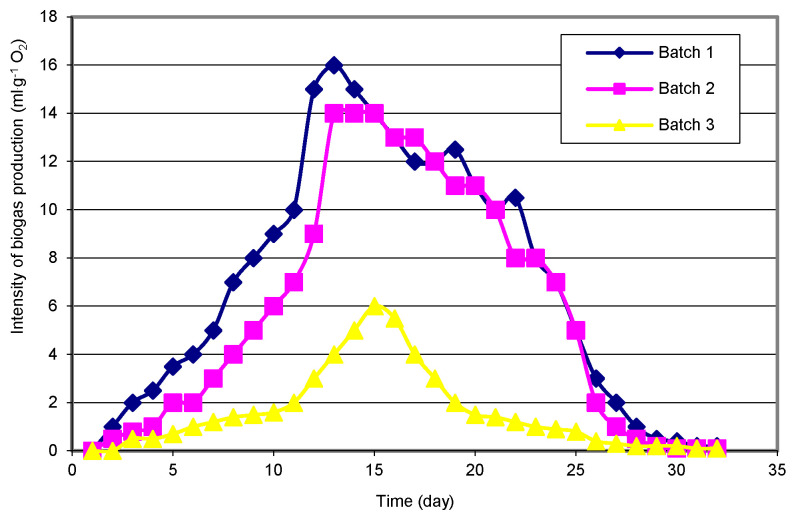
The dynamics of biogas production intensity during the process.

The analysis results indicate a slight variation in the biogas yield for individual research objects. The highest biogas yield, i.e., 404 dm^3^ per 1 kg of dry weight of the batch was observed in the facility with sewage sludge (Figure 1 and Figure 2). Converted into the COD value, this is 0.232 dm^3^·g O_2_ COD. In the case of corn, these values were, respectively, 324 dm^3^ and 0.193 (Figure 1). The lowest yield of biogas was identified in the organic sub-sieve fraction of municipal waste, 287 dm^3^·kg^−1^ dw or 0.178 dm^3^·g O_2_ COD, respectively (Figure 1 and Figure 2). Zhu et al. [48] report the efficiency of municipal waste biogas production at 238–300 dm^3^·kg^−1^ dw of waste, depending on the organic matter load in the fermentation chamber. The results of own research indicate a different pace of the fermentation process (Figure 1 and Figure 2). In the case of sewage sludge and its sub-sieve fraction, an intensive increase in biogas production was observed on the 5th day of the process (Figure 1 and Figure 2). The peak of the process was observed on the 13th day and its decrease, from the 21st day in the case of municipal waste, and the 25th day in the case of sewage sludge (Figure 3). In the case of methane fermentation of corn silage, the initiation of the intensive biogas emission phase was delayed by approximately 5 days compared to the other materials used in the experiment. 

A delay in the process of biogas evolution was observed until day 23 in the medium based on organic fraction of municipal waste, compared with the other fractions studied.

The Tukey test confirmed that there are homogeneous groups of biogas yield in the fermentation of sewage sludge biomass, organic fraction of municipal waste and agricultural biomass in the form of corn silage. Agricultural biomass was characterized by the highest biogas yield and formed a homogeneous group. Sewage sludge biomass and organic fraction of municipal waste formed a homogeneous group, with lower biogas yield during fermentation. The Tukey test confirms that in methane fermentation, agricultural biomass provides better biogas yields than waste biomass.

**Figure 4:**
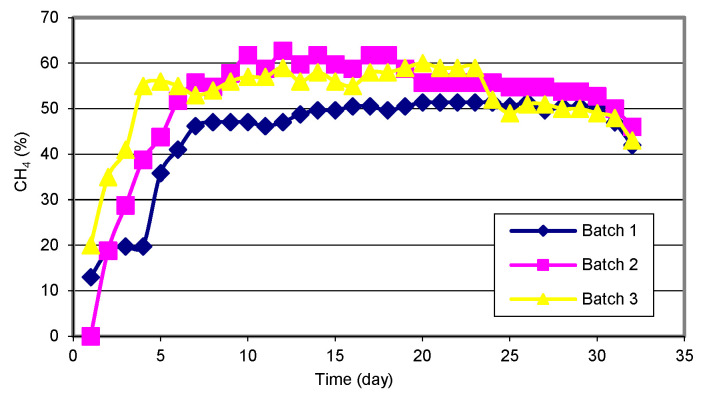
Dynamics of methane (CH_4_) content in the biogas during the process.

The maximum biogas release was confirmed on the 15th day of the process, while from the 28th day of the process, the release of biogas decreased (Figure 3). The methane content is the most important parameter in determining the quality of the produced biogas. The research results show that the highest methane content was obtained in biogas produced from sewage sludge and corn silage. The average content of methane in the biogas produced from these materials was approximately 52% (Figure 4). In the case of both types of waste, the methane content increased until the 7th day of the process, and then its amount stabilized. From the 30th day of the process, a sharp decrease in the methane content in the biogas was observed, and on the 33rd day, its content was 42%. In the case of the sub-sieve fraction of municipal waste, the average methane content was 10% lower compared to the other materials (Figure 4).

**Figure 5:**
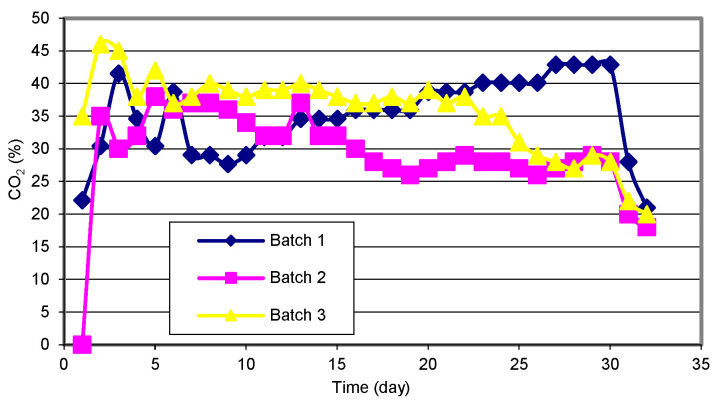
Dynamics of carbon dioxide (CO_2_) content in the biogas during the process.

The dynamics of the changes in this parameter was similar to that observed in corn silage and sewage sludge. The maximum readable methane content, i.e., 61.75% of the volume, was observed on day 12 of the corn silage biogas treatment process. In poultry processing waste biogas, Sikora et al. [50] found the methane content at 75%. In turn, Kymäläinen et al. [51] found that the average content of methane in biogas from the mixture of sewage sludge and municipal waste was at 65%. In turn, Zhu et al. [52] determined approximately 45% methane content in biogas from municipal waste. The dynamics of carbon dioxide content in individual research facilities was similar, regardless of the type of biomass used. Its level in biogas produced from municipal waste was at 35%, while in the case of sewage sludge and corn silage this value was approximately 32% (Figure 5).

The results of own research indicate a slightly lower value of biogas yield in the case of municipal waste compared to corn silage, which was the reference material in the assessment of the suitability of waste materials for biogas production. The reduced efficiency of biogas production from the sub-sieve fraction of municipal waste is related to the nature of this type of waste. Despite the comparable total COD value to COD value found in sewage sludge and corn silage, the key for the methanogenesis process is the value of the soluble COD. In the case of municipal waste, it was 2862 mg O_2_·dm^−3^, and in the case of sewage sludge, this value was approximately 40% higher (Table 2). COD values in the materials used for biogasification were comparable to those reported by other authors. Vu and Min [46] found the value of the total COD for sewage sludge at over 30,000 mg O_2_·dm^−3^, while the value of soluble COD for sewage sludge was found at 4441 mg O_2_·dm^−3^, with a dry weight content of 1.6%. For the digestate, the values reported by these authors were 13,630 mg O_2_·dm^−3^, and 360 mg of O_2_·dm^−3^, respectively.

To increase the production efficiency of the municipal waste methanogenesis process, this waste should be pre-treated. Elalami et al. [53] indicate that the best production results are obtained after microwave treatment of the waste, or by adding alkalizing materials. The authors indicate that a combination of thermal treatment of sewage sludge with a mixture of potassium hydroxide and sodium hydroxide increased the efficiency of methane production by more than 70% compared to facilities using materials that were not treated [54]. Park et al. [55] found an increase in the methanogenesis efficiency in slaughter waste heat-treated at 190 °C. Nguyen et al. [56] indicate that the initial treatment of sewage sludge (physical, thermal, chemical and biochemical) had a positive effect on the processes of methane production. However, the economic efficiency of such processes is difficult to achieve. Sikora et al. [50] report the level of biogas produced from methane fermentation of slaughterhouse waste at 400 cm^3^·g^−1^ dw of waste. Adding 1% of emulsifier increased the efficiency of biogas production and cut the process by approximately 50%. The values of the obtained sewage sludge biogas were comparable to those reported by Sosnowski et al. [57], who found the methane yield from the biogasification of municipal waste at 0.231 dm^3^ CH_4_·g^−1^ of total suspended solids (TSS). For sewage sludge, this value was twice as high. Vu and Min [48] report the methane yield in the fermentation of raw sewage sludge at approximately 0.19 dm^3^ CH_4_·g COD. Electrochemical treatment of sewage sludge increased the methane production efficiency to 0.28 dm^3^ CH_4_·g COD. The results of the conducted analyzes indicate high efficiency of the methane fermentation of the sub-screen fraction of municipal waste, comparable to the results obtained for corn silage. Similarly, Le Hyaric et al. [58] indicate high suitability of the organic fraction of municipal waste for the fermentation process. The authors indicate that a large amount of poorly degradable solid fractions in this material may lead to inhibition of the process. Biogasification of the organic fraction of waste is justified from both economic and environmental perspectives [43]. The problem that hampers the effective utilization of municipal waste is the management of the digestate. Mayer et al. [59] found that the most effective method of municipal waste disposal is biogasification and composting of the resulting digestate. In turn, Kymäläinen et al. [51] found that in terms of the stability of the methanogenesis process, the best results can be obtained when fermenting a mixture of sewage sludge and municipal waste. Sewage sludge contains a large amount of nitrogen, so using it as an ingredient is favorable for the co-fermentation process. Similar conclusions were obtained by Ghosh et al. [60]. These authors emphasize the role of anaerobic fungi in the process of acetogenesis. The use of digestate as a soil improvement agent can be a factor that optimizes the biogas process [4]. 

In terms of environmental efficiency of the biogas production process, it is most advantageous to use digestate for agricultural purposes. The digestate is a by-product of the methane fermentation process and its introduction into the environment is regulated by Polish legislation. The Regulation of the Minister of the Environment on the waste catalog (Journal of Laws No. 112, item 1206, as amended) specifies the code of this type of waste (19 06 06) and classifies it as a digested waste of anaerobic decomposition of animal and vegetable waste. According to the Regulation, it is possible to recover the digestate using the R10 method, i.e., by spreading it over the ground to support cultivation. In organic fertilizers, the permissible content of impurities and the minimum content of fertilizing ingredients are limited, while in agents supporting the cultivation of plants, only the content of impurities is limited [61].

The minimum content of nitrogen, phosphorus, and potassium in organic fertilizers is 3%, 0.684%, and 1.78%, respectively. The maximum content of heavy metals in organic fertilizers is 60 mg Ni·kg^−1^, 100 Cr·kg^−1^, 140 Pb·kg^−1^, and 5 Cd·kg^−1^ [59]. These criteria apply to all organic fertilizers, as well as to aerobically stabilized composts or digestates, as well as purified stabilizers intended to be spread over the ground for fertilization or soil improvement. The results of this research indicate remarkably high nutrient content in the obtained digestates. The nitrogen content in the sewage sludge digestate was 4.339%, calculated per dry weight. In the case of silage, this value was 3.387%, while in the municipal waste digestate, it was 2.881% (Table 3). For all materials, increased nitrogen content was found due to the methanogenesis process. The highest level of phosphorus was found in municipal waste, 3.187 g·kg^−1^ dw per batch on average. In the remaining materials used in the research, the content of phosphorus was approximately 15% lower. The potassium concentration in municipal waste and corn silage was at a similar level, while in sewage sludge its value was more than 10 times higher.

In contrast, Elalami et al. [53,54] found that in the digestate from the fermentation of sewage sludge the nitrogen content was 4.7%. The content of potassium and phosphorus was approximately 15 g·kg^−1^ and 35 g·kg^−1^, respectively. These authors found a positive effect of sewage sludge digestate on the yield and quality of tomatoes. Similarly, Cristina et al. [62] found a positive effect of digestate from sewage sludge on the yielding of tomatoes, but these authors point out the risk of enriching the soil with available forms of heavy metals.

The use of digestate for agricultural purposes is the best method of its utilization, since significant amounts of nutrients return to the biocycle. Intensive cultivation of plants leads to a permanent reduction in the amount of organic matter in soils. Therefore, the introduction of materials containing organic carbon and biogenic elements into the soil is a strategic element of rational agricultural management [63,64]. The use of digestate from municipal waste can be problematic due to possible contamination with a non-degradable fraction [65]. Nevertheless, the potential of using digestate from the methane fermentation of organic municipal waste is currently the most important direction of research on waste disposal in the European Union [66,67,68]. The fermentation process allows microbiological stabilization of post-process waste and its hygienization. This, and the high content of nutrients, allows using its solid fraction for fertilization. Due to the high level of salinity, which may limit their purification processes, managing the liquid part can be problematic [69,70]. Moreover, the author draws attention to the risk of contamination of the digestate with heavy metals. The results of our own research indicate increased chromium content in waste from methanogenesis of organic municipal waste. In the case of sewage sludge, the level of cadmium was above critical for materials introduced into the soil. Its use for fertilization purposes would be associated with the risk of environmental pollution and excessive accumulation of trace elements in food products. The use of waste materials as a means of food production is always associated with risk for the quality of the product [71,72,73,74]. Therefore, waste quality should be controlled regularly, with a frequency consistent with the previously conducted risk analysis.

## 4. Conclusions

The organic fraction of the municipal waste was characterized by approximately 30% lower value of the soluble COD, with a comparable level of total COD compared to other materials. The total amount of biogas obtained in methane fermentation of the organic sub-sieve fraction of municipal waste was 0.179 dm·g^−1^ O_2_ COD. On the other hand, in the case of sewage sludge, this value was 0.193 dm·g^−1^ O_2_ COD. Biogasification of sewage sludge produced biogas at 0.232 dm·g^−1^ O_2_ COD. The type of fermented material did not impact the intensity of biogas production. The maximum level of biogas production occurred between the 13th and 15th day of the process. The digestate obtained in the process of methanogenesis of corn silage and the organic fraction of municipal waste was characterized by good parameters in terms of possible use for fertilization purposes due to high levels of nitrogen, phosphorus and potassium. In the case of sewage sludge, remarkably high cadmium contents were found, which disqualify it for agricultural use.

## Figures and Tables

**Table 1:** Parameters of the analysis method.

Parameters	Sludge	Limit Detection	Content in Certified Material	Measured	Recovery
	(nm)	(mg·dm^−3^)	(mg·kg^−1^)	(mg·kg^−1^)	(%)
Mg	285.208	0.0016	1360	1414.4	104
P	213.617	0.076	2300	2231	97
Ca	317.933	0.01	21,600	22,896	106
Na	589.592	0.069	500	485	97
K	766.490	-	21,000	19,740	94
Cu	327.393	0.0097	9.4	10.058	107
Fe	238.204	0.0046	185	179.45	97
Zn	206.200	0.0059	24	23.52	98
Mn	257.608	0.0014	47	45.84	97.5
Ni	231.604	0.0151	4	3.89	97.3
Pb	220.353	0.0425	1.6	1.544	96.5
Cr	267.707	0.0071	6.5	6.96	107.1
Cd	228.802	0.0027	0.03	0.0311	103.7

**Table 2:** Basic parameters of materials used in the methanogenesis process.

Type of Material	Dry Weight Content	pH	Volatile Suspended Solids (VSS)	Mineral Substances	Organic Substances	COD Value (Total)	COD Value (Soluble)
	(%)	-	(%)	(%)	(%)	(mg O_2_·dm^−1^)	(mg O_2_·dm^−1^)
Sludge (Batch 1)	1.8	6.0	1.7	21.7	68.5	29,582	4023
Organic fraction of municipal waste (Batch 2)	1.8	6.9	1.9	19.4	79.72	30,523	2862
Corn silage (Batch 3)	1.8	3.8	1.85	23.4	85.6	30,455	3846

**Table 3:** Chemical composition of the input material and digestate.

Parameters	Sewage Sludge	Municipal Waste	Silage	Sewage Sludge	Municipal Waste	Silage
	Digestate			Input Material		
	(%)					
C	38.56	41.89	47.28	65.55	51.46	75.18
N	4.339	2.881	3.387	5.484	2.615	2.845
C:N	8.887	14.54	13.96	11.95	19.68	26.42
(g·kg^−1^)						
Mg	2.542	3.187	2.495	3.354	3.520	2.121
P	2.542	3.187	2.495	3.254	3.520	2.821
Ca	35.21	28.58	17.39	39.34	26.42	17.22
Na	30.06	43.17	44.92	39.67	47.67	38.18
K	0.980	11.53	12.34	1.207	13.27	10.12
(mg·kg^−1^)						
Cu	83.01	47.68	33.68	109.5	52.65	28.63
Fe	4857	2438	767.7	5854	2615	621.8
Zn	295.9	142.5	94.49	380.7	153.2	82.21
Mn	173.1	80.42	96.43	228.4	88.81	81.96
Ni	26.51	3.227	2.474	34.98	3.564	2.103
Pb	9.856	3.114	1.038	14.88	3.967	0.779
Cr	27.515	4.693	−0.053	36.31	5.183	−0.045
Cd	9.521	0.307	0.269	14.91	0.407	0.195

## Data Availability

Not applicable.

## References

[1] Lesovik V.S., Zagorodnyuk L.K., Babaev Z.K., Dzhumaniyazov Z.B. (2020). Analysis of the Causes of Brickwork Efflorescence in the Aral Sea Region. Glas. Ceram..

[2] Murali G., Abid S.R., Amran Y.M., Abdelgader H.S., Fediuk R., Susrutha A., Poonguzhali K. (2020). Impact performance of novel multi-layered prepacked aggregate fibrous composites under compression and bending. Structures.

[3] Kolesnikov A.S., Kenzhibaeva G.S., Botabaev N.E., Kutzhanova A.N., Iztleuov G.M., Suigenbaeva A.Z., Ashirbekov K.A., Kolesnikova O.G. (2020). Thermodynamic Modeling of Chemical and Phase Transformations in a Waelz Process-Slag—Carbon System. Refract. Ind. Ceram..

[4] Sikora J., Niemiec M., Szelag-Sikora A. (2018). Evaluation of the chemical composition of raw common duckweed (*Lemna minor* L.) and pulp after methane fermentation. J. Elem..

[5] Kasprzak K., Wojtunik-Kulesza K., Oniszczuk T., Kubon M., Oniszczuk A. (2018). Secondary Metabolites, Dietary Fiber and Conjugated Fatty Acids as Functional Food Ingredients against Overweight and Obesity. Nat. Prod. Commun..

[6] Szparaga A., Kubon M., Kocira S., Czerwińska E., Pawłowska A., Hara P., Kobus Z., Kwaśniewski D. (2019). Towards Sustainable Agriculture—Agronomic and Economic Effects of Biostimulant Use in Common Bean Cultivation. Sustainability.

[7] Tryhuba A., Hutsol T., Tryhuba I., Pokotylska N., Kovalenko N., Tabor S., Kwasniewski D. (2021). Risk Assessment of Investments in Projects of Production of Raw Materials for Bioethanol. Processes.

[8] Mangwandi C., Tao L.J., Albadarin A.B., Allen S.J., Walker G.M. (2013). Alternative method for producing organic fertiliser from anaerobic digestion liquor and limestone powder: High Shear wet granulation. Powder Technol..

[9] Sikora J., Niemiec M., Szelag-Sikora A., Kuboń M., Olech E., Marczuk A. (2017). Biogasification of wastes from industrial processing of carps. Przem. Chem..

[10] Popardowski E., Kwaśniewski D. (2017). Technical-economic aspects of the eradication of energy willow plantations. MendelNet.

[11] Mudryk K., Jewiarz M., Wróbel M., Niemiec M., Dyjakon A. (2021). Evaluation of urban tree leaf biomass-potential, physico-mechanical and chemical parameters of raw material and solid biofuel. Energies.

[12] Lutsiak V., Hutsol T., Kovalenko N., Kwaśniewski D., Kowalczyk Z., Belei S., Marusei T. (2021). Enterprise Activity Modeling in Walnut Sector in Ukraine. Sustainability.

[13] Alkanok G., Demirel B., Onay T.T. (2014). Determination of biogas generation potential as a renewable energy source from supermarket wastes. Waste Manag..

[14] De Souza S.N.M., Wernacke I., Marques C.A., Bariccatti R.A., Santos R.F.S., Nogueira C.E.C., Bassegio D. (2013). Electric energy micro-production in a rural property using biogas as primary source. Renew. Sust. Energ. Rev..

[15] Lijó L., González-García S., Bacenetti J., Moreira M.T. (2017). The environmental effect of substituting energy crops for food waste as feedstock for biogas production. Energy.

[16] Aravani V.P., Sun H., Yang Z., Liu G., Wang W., Anagnostopoulos G., Syriopoulos G., Charisiou N.D., Goula M.A., Kornaros M. (2021). Agricultural and livestock sector’s residues in Greece & China: Comparative qualitative and quantitative characterization for assessing their potential for biogas production. Renew. Sust. Energy Rev..

[17] Yoshida H., Tokumoto H., RyoIshii K. (2009). Efficient, high-speed methane fermentation for sewage sludge using subcritical water hydrolysis as pretreatment. Bioresour. Technol..

[18] Loganath R., Mazumder D. (2018). Performance study on organic carbon, total nitrogen, suspended solids removal and biogas production in hybrid UASB reactor treating real slaughterhouse wastewater. J. Environ. Chem. Eng..

[19] Pafili A., Charisiou N.D., Douvartzides S.L., Siakavelas G.I., Wang W., Liu G., Papadakis V.G., Goula M.A. (2021). Recent Progress in the Steam Reforming of Bio-Oil for Hydrogen Production: A Review of Operating Parameters, Catalytic Systems and Technological Innovations. Catalysts.

[20] Jung S., Lee J., Moon D.H., Kim K.-H., Kwon E.E. (2021). Upgrading biogas into syngas through dry reforming. Renew. Sustain. Energy Rev..

[21] Kuboń M., Krasnodębski A. (2010). Logistic cost in competitive strategies of enterprises. Agric. Econ..

[22] Szeląg-Sikora A., Sikora J., Niemiec M., Gródek-Szostak Z., Kapusta-Duch J., Kuboń M., Komorowska M., Karcz J. (2019). Impact of Integrated and Conventional Plant Production on Selected Soil Parameters in Carrot Production. Sustainability.

[23] Niemiec M., Komorowska M., Szeląg-Sikora A., Sikora J., Kuboń M., Gródek-Szostak Z., Kapusta-Duch J. (2019). Risk Assessment for Social Practices in Small Vegetable farms in Poland as a Tool for the Optimization of Quality Management Systems. Sustainability.

[24] Korys K.A., Latawiec A.E., Grotkiewicz K., Kubon M. (2019). The Review of Biomass Potential for Agricultural Biogas Production in Poland. Sustainability.

[25] Lorenz H., Fischer P., Schumacher B., Adler P. (2013). Current EU-27 technical potential of organic waste streams for biogas and energy production. Waste Manag..

[26] Tylek P., Pietrzykowski M., Walczyk J., Juliszewski T., Kwaśniewski D. (2017). Root Biomass and Morphological Characterization of Energy Willow Stumps. Croat. J. For. Eng..

[27] Granada C.E., Hasan C., Marder M., Konrad O., Vargas L.K., Passaglia L.M.P., Giongo A., de Oliveira R.R., de Pereira M.L., Trindade F.J. (2018). Biogas from slaughterhouse wastewater anaerobic digestion is driven by the archaeal family Methanobacteriaceae and bacterial families Porphyromonadaceae and Tissierellaceae. Renew. Energy.

[28] Gródek-Szostak Z., Luc M., Szeląg-Sikora A., Sikora J., Niemiec M., Ochoa Siguencia L., Velinov E. (2020). Promotion of RES in a Technology Transfer Network. Case Study of the Enterprise Europe Network. Energies.

[29] Velinov E., Petrenko Y., Vechkinzova E., Denisov I., Ochoa Siguencia L., Gródek-Szostak Z. (2020). “Leaky Bucket” of Kazakhstan’s Power Grid: Losses and Inefficient Distribution of Electric Power. Energies.

[30] Gródek-Szostak Z., Suder M., Kusa R., Szeląg-Sikora A., Duda J., Niemiec M. (2020). Renewable Energy Promotion Instruments Used by Innovation Brokers in a Technology Transfer Network. Case Study of the Enterprise Europe Network. Energies.

[31] Rashidov N., Chowaniak M., Niemiec M., Mamurovich G.S., Gufronovich M.J., Gródek-Szostak Z., Szeląg-Sikora A., Sikora J., Kuboń M., Komorowska M. (2021). Assessment of the Multiannual Impact of the Grape Training System on GHG Emissions in North Tajikistan. Energies.

[32] Pietrzykowski M., Woś B., Tylek P., Kwaśniewski D., Juliszewski T., Walczyk J., Likus-Cieślik J., Ochał W., Tabor S. (2021). Carbon sink potential and allocation in above- and below-ground biomass in willow coppice. J. For. Res..

[33] Vilalba G., Liu Y., Schroder H., Ayres R.U. (2008). Global phosphorus flows in the industrial economy from a production perspective. J. Ind. Ecol..

[34] Weiland P. (2006). Biomass digestion in agriculture: A successfull pathway for the energy production and waste treatment in Germany. Eng. Life Sci..

[35] Niemiec M., Komorowska M., Szeląg-Sikora A., Sikora J., Kuzminova N. (2018). Content of Ba, B, Sr and As in water and fish larvae of the genus Atherinidae L. sampled in three bays in the Sevastopol coastal area. J. Elem..

[36] Kwaśniewski D., Płonka A., Mickiewicz P. (2022). Harvesting Technologies and Costs of Biomass Production from Energy Crops Cultivated on Farms in the Małopolska Region. Energies.

[37] Jurgutis L., Šlepetienė A., Amalevičiūtė-Volungė K., Volungevičius J., Šlepetys J. (2021). The effect of digestate fertilisation on grass biogas yield and soil properties in field-biomass-biogas-field renewable energy production approach in Lithuania. Biomass Bioenergy.

[38] Mingjing S.D., Xiong H.X., Tsang D.C.W. (2021). Sustainable management and recycling of food waste anaerobic digestate: A review. Bioresour. Technol..

[39] Niemiec M., Szeląg-Sikora A., Cupiał M. (2015). Evaluation of the Efficiency of Celeriac Fertilization with the Use of Slow-Acting Fertilizers.

[40] Gondek K., Mierzwa-Hersztek M., Kopeć M., Sikora J., Lošák T., Grzybowski P. (2019). Sewage Sludge Biochar Effects on Phosphorus Mobility in Soil and Accumulation in Plant. Ecol. Chem. Eng. S.

[41] Gondek K., Mierzwa-Hersztek M., Kopeć M., Sikora J., Głąb T., Szczurowska K. (2019). Influence of Biochar Application on Reduced Acidification of Sandy Soil, Increased Cation Exchange Capacity, and the Content of Available Forms of K, Mg, and P. Pol. J. Environ. Stud..

[42] Szeląg-Sikora A., Niemiec M., Sikora J., Chowaniak M., Lorencowicz E., Uziak J., Huyghebaert B. (2017). Possibilities of Designating Swards of Grasses and Small-Seed Legumes from Selected Organic Farms in Poland for Feed, W: Farm Machinery and Processes Management in Sustainable Agriculture: IX International Scientific Symposium Symposium Proceedings.

[43] Council Directive 1999/31/EC of 26 April 1999 on the Landfill of Waste. https://eur-lex.europa.eu/legal-content/EN/TXT/?uri=CELEX%3A31999L0031.

[44] Regulation (EC) No 1069/2009 of the European Parliament and of the Council of 21 October 2009 Laying Down Health Rules as Regards Animal By-Products and Derived Products not Intended for Human Consumption and Repealing Regulation (EC) No 1774/2002 (Animal By-Products Regulation). https://eur-lex.europa.eu/legal-content/en/ALL/?uri=CELEX%3A32009R1069.

[45] The Act of 14 December 2012 on Waste. Journal of Laws of 2013, Item 21. https://www.global-regulation.com/translation/poland/8302260/act-of-14-december-2012-on-waste.html.

[46] Latifi P., Karrabi M., Danesh S. (2019). Anaerobic co-digestion of poultry slaughterhouse wastes with sewage sludge in batch-mode bioreactors (effect of inoculum-substrate ratio and total solids). Renew. Sustain. Energy Rev..

[47] Romano R.T., Zhang R.H. (2008). Co-digestion of onion juice and wastewater sludge using an anaerobic mixed biofilm reactor. Bioresour. Technol..

[48] Vu H.T., Min B. (2019). Enhanced methane fermentation of municipal sewage sludge by microbial electrochemical systems integrated with anaerobic digestion. Int. J. Hydrog. Energy.

[49] Choi K.-S., Kondaveeti S., Min B. (2017). Bioelectrochemical methane (CH4) production in anaerobic digestion at different supplemental voltages. Bioresour. Technol..

[50] Sikora J., Niemiec M., Szeląg-Sikora A., Gródek-Szostak Z., Kuboń M., Komorowska M. (2020). The Effect of the Addition of a Fat Emulsifier on the Amount and Quality of the Obtained Biogas. Energies.

[51] Kymäläinen M., Lähde K., Arnold M., Kurola J., Romantschuk M., Kautola H. (2012). Biogasification of biowaste and sewage sludge—Measurement of biogas quality. J. Environ. Manag..

[52] Zhu B., Zhang R., Gikas P., Rapport J., Jenkins B., Li X. (2010). Biogas production from municipal solid wastes using an integrated rotary drum and anaerobic-phased solids digester system. Bioresour. Technol..

[53] Elalami D., Carrere H., Abdelouahdi K., Garcia-Bernet D., Peydecastaing J., Vaca-Medina G., Oukarroum A., Zeroual Y., Barakat A. (2020). Mild microwaves, ultrasonic and alkaline pretreatments for improving methane production: Impact on biochemical and structural properties of olive pomace. Bioresour. Technol..

[54] Elalami D., Monlau F., Carrere H., Abdelouahdi K., Oukarroum A., Zeroual Y., Barakat A. (2020). Effect of coupling alkaline pretreatment and sewage sludge co-digestion on methane production and fertilizer potential of digestate. Sci. Total Environ..

[55] Park S., Yoon Y.-M., Han S.K., Kim D., Kim H. (2017). Effect of hydrothermal pre-treatment (HTP) on poultry slaughterhouse waste (PSW) sludge for the enhancement of the solubilization, physical properties, and biogas production through anaerobic digestion. Waste Manag..

[56] Nguyen V.K., Chaudhary D.K., Dahal R.H., Trinh N.H., Kim J., Chang S.W., Hong Y., La D.D., Nguyen X.C., Ngo H.H. (2021). Review on pretreatment techniques to improve anaerobic digestion of sewage sludge. Fuel.

[57] Sosnowski P., Klepacz-Smolka A., Kaczorek K., Ledakowicz S. (2008). Kinetic investigations of methane co-fermentation of sewage sludge and organic fraction of municipal solid wastes. Bioresour. Technol..

[58] Le Hyaric R., Chardin C., Benbelkacem H., Bollon J., Bayard R., Escudié R., Buffière P. (2011). Influence of substrate concentration and moisture content on the specific methanogenic activity of dry mesophilic municipal solid waste digestate spiked with propionate. Bioresour. Technol..

[59] Mayer F., Bhandari R., Gäth S.A., Himanshu H., Stobernack N. (2020). Economic and environmental life cycle assessment of organic waste treatment by means of incineration and biogasification. Is source segregation of biowaste justified in Germany?. Sci. Total Environ..

[60] Ghosh P., Kumar M., Kapoor R., Kumar S.S., Singh L., Vijay V., Vijay V.K., Kumar V., Thakur I.S. (2020). Enhanced biogas production from municipal solid waste via co-digestion with sewage sludge and metabolic pathway analysis. Bioresour. Technol..

[61] (2008). Regulation of the Minister of Agriculture and Rural Development, of 18 June 2008, on execution of some regulations of of act on fertilizers and on fertilizing. J. Law.

[62] Cristina G., Camelin E., Tommasi T., Fino D., Pugliese M. (2020). Anaerobic digestates from sewage sludge used as fertilizer on a poor alkaline sandy soil and on a peat substrate: Effects on tomato plants growth and on soil properties. J. Environ. Manag..

[63] Niemiec M., Chowaniak M., Sikora J., Szeląg-Sikora A., Gródek-Szostak Z., Komorowska M. (2020). Selected Properties of Soils for Long-Term Use in Organic Farming. Sustainability.

[64] Sikora J., Niemiec M., Tabak M., Gródek-Szostak Z., Szeląg-Sikora A., Kuboń M., Komorowska M. (2020). Assessment of the efficiency of nitrogen slow-release fertilizers in integrated production of carrot depending on fertilization strategy. Sustainability.

[65] Cesaro A. (2020). The valorization of the anaerobic digestate from the organic fractions of municipal solid waste: Challenges and perspectives. J. Environ. Manag..

[66] Somers M.H., Azman S., Sigurnjak I., Ghyselbrecht K., Meers E., Meesschaert B., Appels L. (2018). Effect of digestate disintegration on anaerobic digestion of organic waste. Bioresour. Technol..

[67] Stürmer B., Pfundtner E., Kirchmeyr F., Uschnig S. (2020). Legal requirements for digestate as fertilizer in Austria and the European Union compared to actual technical parameters. J. Environ. Manag..

[68] Lotti T., Burzi O., Scaglione D., Ramos C.A., Ficara E., Pérez J., Carrera J. (2019). Two-stage granular sludge partial nitritation/anammox process for the treatment of digestate from the anaerobic digestion of the organic fraction of municipal solid waste. Waste Manag..

[69] Bustamante M.A., Restrepo A.P., Alburquerque J.A., Pérez-Murcia M.D., Paredes C., Moral R., Bernal M.P. (2013). Recycling of anaerobic digestates by composting: Effect of the bulking agent used. J. Clean. Prod..

[70] Kowalczyk Z., Kwaśniewski D. (2021). Environmental impact of the cultivation of energy willow in Poland. Sci. Rep..

[71] Lisowska A., Filipek-Mazur B., Sołtys J., Niemiec M., Gorczyca O., Bar-Michalczyk D., Komorowska M., Gródek-Szostak Z., Szeląg-Sikora A., Sikora J. (2022). Preparation, Characterization of Granulated Sulfur Fertilizers and Their Effects on a Sandy Soils. Materials.

[72] Tryhuba A., Hutsol T., Głowacki S., Tryhuba I., Tabor S., Kwaśniewski D., Sorokin D., Yermakov S. (2021). Forecasting Quantitative Risk Indicators of Investors in Projects of Biohydrogen Production from Agricultural Raw Materials. Processes.

[73] Jung S., Shetti N.P., Reddy K.R., Nadagouda M.N., Park Y.-K., Aminabhavi T.M., Kwon E.E. (2021). Synthesis of different biofuels from livestock waste materials and their potential as sustainable feedstocks—A review. Energy Convers. Manag..

[74] Gao Y., Jianga J., Meng Y., Yan F., Aihemaitia A. (2018). A review of recent developments in hydrogen production via biogas dry reforming. Energy Convers. Manag..

